# Integration of comparative transcriptomics and WGCNA characterizes the regulation of anthocyanin biosynthesis in mung bean (*Vigna radiata* L.)

**DOI:** 10.3389/fpls.2023.1251464

**Published:** 2023-10-24

**Authors:** Chunxia Li, Zexiang Gao, Weili Hu, Xu Zhu, Youjun Li, Na Li, Chao Ma

**Affiliations:** ^1^ College of Agriculture, Henan University of Science and Technology, Luoyang, China; ^2^ Dry-land Agricultural Engineering Technology Research Center in Henan, Henan University of Science and Technology, Luoyang, Henan, China; ^3^ Crop Breeding Research Center, Nanyang Academy of Agricultural Science, Nanyang, Henan, China

**Keywords:** mung bean, anthocyanins, transcriptome sequencing, weighted gene co-expression analysis, differentially expressed genes

## Abstract

Mung bean is a dual-use crop widely cultivated in Southeast Asia as a food and medicine resource. The development of new functional mung bean varieties demands identifying new genes regulating anthocyanidin synthesis and investigating their molecular mechanism. In this study, we used high-throughput sequencing technology to generate transcriptome sequence of leaves, petioles, and hypocotyls for investigating the anthocyanins accumulation in common mung bean variety as well as anthocyanidin rich mung bean variety, and to elucidate their molecular mechanisms. 29 kinds of anthocyanin compounds were identified. Most of the anthocyanin components contents were significantly higher in ZL23 compare with AL12. Transcriptome analysis suggested that a total of 93 structural genes encoding the anthocyanin biosynthetic pathway and 273 regulatory genes encoding the ternary complex of MYB-bHLH-WD40 were identified, of which 26 and 78 were differentially expressed in the two varieties. Weighted gene co-expression network analysis revealed that VrMYB3 and VrMYB90 might have enhanced mung bean anthocyanin content by inducing the expression of structural genes such as *PAL*, *4CL*, *F3’5’H*, *LDOX*, and *F3’H*, which was consistent with qRT-PCR results. These findings are envisaged to provide a reference for studying the molecular mechanism of anthocyanin accumulation in mung beans.

## 1 Introduction

Mung bean (*Vigna radiata* L.) is a cowpea (*Vigna*) plant belonging to the leguminosae family and is one of the fastest-growing edible legume crops in Asia (Han and Na, 2021; Zhao et al., 2022). Not only is it drought- and infertile-resistant, and easy to cultivate, it can also symbiotically fix nitrogen, making it a better alternative to cereals, sweet potatoes, cotton and other fruit trees. (Ilyas et al., 2018; Qian et al., 2018; Gong et al., 2020). It has a high nutritional values as it is rich in amino acids, vitamins, proteins, and minerals, and also contains many flavonoids with antioxidant and anti-aging properties. As an important source of protein supplements, mung bean has become a crucial part of human food consumption, especially in financially vulnerable regions (Luo et al., 2016; Zhu et al., 2018; Kumar and Pandey, 2020). Continuous improvement in living standards and increasing health awareness have created the need for diversified nutritious and healthy pulses, and this has also become a new target for the mung bean. More importantly, the color of cereal products has become an important indicator of the nutritional content and economic value of these products (Thakur S, et al., 2019; Li et al., 2022a).

Anthocyanins, which are water-soluble pigments found in leaves, fruits, grains, and flowers, impart various colors to plants including red, blue, or purple (Leyrer et al., 2016). Following different modifications, biosynthetic anthocyanins are transported to vesicles and other locations for storage (Masukawa et al., 2018). In plants, anthocyanins have been reported to possess diverse biological functions such as scavenging free radicals generated during physiological metabolism under biotic or abiotic stress conditions. This helps reduce cell damage and maintain normal photosynthetic activity (Li et al., 2022b). Additionally, anthocyanins contribute to the vibrant colors of plant flowers and aid in attracting insects for pollination purposes (Wim and El-Esawe, 2014). As phytonutrients with potent antioxidant and anti-mutagenic activities, they play a pivotal role in human health (Li et al., 2019b; Yousuf et al., 2016). As one of the important secondary metabolites and bioactive substances in mung beans, anthocyanins have become one of the hot spots in the current research on the development of functional mung beans (Ma et al., 2023). They are considered major antioxidants along with numerous other compounds present in mung beans (Kan et al., 2018). The biosynthesis of anthocyanins is regulated by two major groups of structural genes, namely early and late biosynthetic genes. The former comprises includes *chalcone synthase* (*CHS*), *chalcone isomerase* (*CHI*), *flavanone 3-hydroxylase* (*F3H*), *flavonoid 3’-hydroxylase* (*F3’H*), and *flavonoid 3’5’-hydroxylase* (*F3’5’H*), which are responsible for the synthesis of flavonols and flavonoids (Stotz et al., 1985; Wienand et al., 1986; Tunen et al., 1988; Meldgaard, 1992; Tanaka et al., 1996). The latter comprises *leucoanthocyanidin dioxygenase* (*LDOX*), *anthocyanidin synthase* (*ANS*), and *UDP-flavonoid glycosyltransferase* (*UFGT*) (Heller et al., 1985; Ju et al., 1995; Simoneau, 1999). Studies investigating the regulation of anthocyanins have primarily focused on MYB, bHLH, and WD40 transcription factors, which form a ternary complex that regulates the expression of structural genes involved in anthocyanin biosynthesis (Shi et al., 2019). Furthermore, various environmental factors can indirectly influence anthocyanin content (Gao et al., 2021). Several studies have demonstrated that light, temperature, low phosphorus levels, and hormones can regulate the expression of anthocyanin synthesis genes (Yang et al., 2017; Saigo et al., 2020; Mo et al., 2021).

Recent studies utilizing high-throughput sequencing technology and multi-omics research methods have revealed that transcription factors such as NAC, bZIP, MADS-box, and GRF-like are also involved in regulating anthocyanin biosynthesis besides MYB-, bHLH-, and WD40-proteins. This expanded understanding is anticipated to explain significant variations observed in anthocyanin profiles among different plant species (Rouholamin et al., 2015; Deng et al., 2020; Bao et al., 2022; Fu et al., 2022). Extensive elucidation has been made regarding the biosynthetic pathways of anthocyanins in crops, such as wheat, maize, potato, and sweet potato (Jiang et al., 2018; Hu et al., 2020; Qi et al., 2023; Fu et al., 2022). However, the accumulation and distribution patterns of anthocyanins in mung bean, as well as the gene regulatory network of biosynthesis, remain unexplored. Therefore, it is necessary to study the molecular mechanism of anthocyanin synthesis in mung bean. In this study, total anthocyanin content in leaves, petioles, and hypocotyls was analyzed using common (AL12), and anthocyanidin-rich mung bean variety (ZL23), where key genes regulating anthocyanin synthesis were identified by multiple omics combined with WGCNA analysis. The results of this study not only extend our understanding of the biosynthesis of anthocyanin-like compounds in mung beans at the molecular level but also provide valuable information for the further development of novel mung beans based nutraceuticals.

## 2 Materials and methods

### 2.1 Plant materials

AL12 (almost no anthocyanidin in the aboveground organs) and ZL23 (ZL23, anthocyanidin rich in leaves, petioles and hypocotyls) varieties of mung beans were used as plant materials whose seeds were generously provided by the Miscellaneous Grains Research Laboratory of Henan University of Science and Technology. The experiment was carried out from May to September 2022. All experimental procedures on plant materials were conducted at the experimental farm of Henan University of Science and Technology, situated at 34° 41′ N and 112° 27′ E, at an altitude of 280 m above sea level. The mean annual rainfall and temperature in the region is 578.2 mm and 14.8°C, respectively. The soil in the experimental farm is classified as fluvo-aquic soil, and has the following physicochemical characteristics: pH (H_2_O) = 7.08, available nitrogen = 80.09 mg·kg^–1^, available phosphorus = 3.31 mg·kg^–1^, available potassium = 81.32 mg·kg^–1^, organic matter = 14.5 g·kg^–1^. Cultivation, and cultivation management was kept the same as for the general high-yielding fields. The leaves, petioles, and hypocotyls of both varieties were sampled after the 5-leaves stages. GL, GB, and GP stand for leaves, petioles, and hypocotyls of AL12, respectively; BL, BB, and BP stand for leaves, petioles, and hypocotyls of ZL23, respectively. Samples were snap frozen in liquid nitrogen and stored at -80°C in a refrigerator till further use. In order to ensure the reliability and reproducibility of the experiment, physiological indexes, metabolome analysis and transcriptome analysis both use 3 biological replicates.

### 2.2 Total anthocyanin measurement and metabolite profiling analysis

The total anthocyanin contents were determined using the hydrochloric acid (HCl)-ethanol method (Wang et al., 2013). Briefly, an accurately weighed 0.1g of sample was taken and ground using a tissue grinder in liquid nitrogen into powder and extracted at 60°C in a water bath for 30 min with 10 ml of 0.1 mol/L HCl-ethanolic solution. The supernatant was carefully separated and transferred to a new 25 ml volumetric flask and extracted again as per the previous procedure. Both the extract fractions were combined, and the final volume was made up to 25 ml using HCl-ethanolic solution. The absorbance of the extract was then recorded at wavelengths of 530 nm, 620nm, and 650nm, respectively. The anthocyanin contents were then estimated using the following equation, and worked out their content by molecular weight of cyanidin-3-O-galactoside.


(1) $${\text{OD}}_{\text{λ}}=(\text{OD}530-\text{OD}620)-0.1(\text{OD}650-\text{OD}620)$$

Where OD is optical density.


(2) $$\text{Content }(\text{mg}/\text{g})\;=\;\frac{{\text{OD}}_{\text{λ}}}{\text{ε}}\times \frac{\text{V}}{\text{m}}\times \frac{1}{1000}\times \text{ M}$$

Where V is total volume of extract; ε is molar extinction coefficient of anthocyanin; m is sample weight; M is molecular weight of cyanidin-3-O-galactoside (449 g/mol).

Anthocyanin metabolome analysis was completed by Wuhan Metware Biotechnology Co., Ltd. through LC-MS/MS. Lyophilized plant tissue sample was ground into powder by a tissue grinder (MM 400, Retsch). 50 mg sample was dissolved in 500 μL extract solution (50% methanol containing 0.1% HCl). The extract solution was oscillated by vortices for 5 min, and was extracted by ultrasonic extractor for 5 min. Then, the extract solution was centrifuged for 10 min (4°C, 12000 rpm min^-1^). The supernatant was transferred to a new centrifuge tube and repeat the extraction process. The extract was filtered with a microporous filter membrane (0.22μm pore size). The extract was then analyzed using a liquid chromatography tandem mass spectrometry system (Applied Biosystems 6500 QTRAP). The liquid chromatographic conditions were as follows: the column was C18 (ACQUITY BEH, 1.7μm, 2.1mm×100mm), and the mobile phase A was ultrapure water (containing 0.1% formic acid), B was methanol (containing 0.1% formic acid), the flow rate was 0.35 mL min^-1^, the column temperature was 40°C, and the injection was 2 μL. Mass spectrometry conditions were as follows: electrospray ion source temperature was 550°C, mass spectrometry voltage was 5500V, curtain gas was 35psi. The acquired mass spectrometry data were then qualitatively analyzed by means of a local database. Finally, the anthocyanin components were quantitatively analyzed using the multiple reaction monitoring mode of triple quadrupole mass spectrometry.

### 2.3 RNA extraction, library construction, and sequencing

Total RNA was extracted from samples using TaKaRa MiniBEST Plant RNA Extraction Kit (TAKARA 9769), and cDNA libraries were constructed for samples meeting the purity, concentration, and integrity of the RNA. The mRNA was enriched with magnetic beads with Oligo (dT), and randomly interrupted by adding a fragmentation buffer. The first cDNA strand was synthesized with random hexamers using the mRNA as a template, followed by purification through AMPure XP beads. The purified double-stranded cDNA was then end-repaired, A-tailed, and ligated to sequencing junctions, followed by fragment size selection using AMPure XP beads, and finally enriched by PCR to obtain the cDNA library. The cDNA libraries were sequenced by Biomarker Technologies Ltd. (Beijing, China) using the Illumina platform. The adapter sequences and low-quality sequences of the off-machine data were filtered to obtain clean data. Sequence alignment was performed with the reference genome of mung bean, and uniq mapped genes were obtained through HISAT2 and StringTie software. Functional annotation of a single gene was performed using multiple databases including NR (Non-Redundant Protein Sequence Database), Swiss-Prot (Swiss Protein Sequence Database), GO (Gene Gntology Annotation), COG (Clusters of Orthologous Groups), KOG (EuKaryotic Orthologous Groups), Pfam (Protein Family Analysis and Modeling), and KEGG (Kyoto Encyclopedia of Genes and Genomes).

### 2.4 Differentially expressed genes and WGCNA

The expression levels of all genes were calculated and normalized to fragments per kilobase of transcript per million fragments mapped (FPKM). Fold Change≥1 and FDR<0.05 were used as the criteria for screening differential expression genes (DEGs). The identified DEGs were further enriched by GO, COG, and KEGG pathway analysis. Gene co-expression network analysis was constructed using the R language software WGCNA package. Since input gene expression was the result of TPM normalization, the expression was first subjected to a further log2 transformation. To ensure that the data conform to the scale-free network distribution, the weighting coefficient β value was screened and the network topology was analyzed using the pickSoftThreshold function in the WGCNA package to obtain the correlation value (R^2^) corresponding to the alternative soft threshold, which is required to ensure R^2^ is close to 0.8 having a certain degree of gene connectivity. A horizontal line was drawn with R^2 = ^0.8 as the threshold, and the first-time power exceeding the threshold was found to be 15. Since power=15 was having a high degree of connectivity, the β=15 was selected to construct the co-expression network and for subsequent analysis. To quantify the co-expression similarity of the whole module, the dynamic tree cut algorithm was used to identify the co-expression modules of the transcriptome expression data, and the minimum number of variables allowed to be included in the module (minModuleSize) was set to 100. To quantify the similarity of co-expression across modules, the characteristic genes of the modules were calculated, with unmerged modules, and other parameters were set by default. The modules are plotted below the tree using the plot dendro and colors function. The soft connectivity function was used to calculate the connectivity between genes, and the top 10 genes with kME values as the core genes of the module (hubgene) were selected. The topological overlap matrix was used to calculate the weights of the different genes in the module, where a higher weight value indicated a higher degree of association between genes. Due to the large network size, a weight value > 0.35 was set as network filtering criteria, and the reciprocal network of 10 hubgene was visualized using Cytoscape v3.6.1. Based on the results of WGCNA, the dark green gene module with the highest correlation to anthocyanins was selected, and the genes within the module were further visualized and analyzed using Cytoscape v3.6.1 for the interactions network. The top 8 genes with the highest degree of linkage were selected as the core genes (hubgene) and were focused owing to key genes affecting anthocyanin biosynthesis.

### 2.5 Gene expression analysis by qRT-PCR

Total RNA extraction, reverse transcription reactions, and qPCR were performed using the kit method (TaKaRa; 9769, RR047B, and RR430B), and primers were designed (**Table S1**). A total of 20 µl reaction volume contained 10 µl of TB Green Fast qPCR Mix (2×), 1 µl cDNA, 0.4 µl each of upstream and downstream primers (10 μmol. L^-1^), and 8.2 µl doubled distilled water (ddH_2_O). The reaction proceeded in a two-step process i.e., pre-denaturation at 95°C for 30 seconds, followed by denaturation at 95°C for 5 seconds, and annealing at 60°C for 30 seconds. A total of 40 cycles were performed, and fluorescence was determined at reversion time. The experiment was conducted in triplicates and relative expression was calculated using the 2^-^ *^ΔΔCT^* method with *VrActin* as the internal reference gene.

### 2.6 Subcellular localization of MYB3 and MYB90

The CDS sequences of MYB3 and MYB90 were cloned into *pCAMBIA1300-GFP* vector without termination. Recombinant plasmid of *35S:MYB3-GFP* and *35S:MYB90-GFP* were transformed into agrobacterium GV3101 for subcellular localization. Agrobacterium (35S:VrMYB3-GFP and 35S:VrMYB90-GFP) was identified as positive clones which was cultured with liquid LB medium (50 μg mL^-1^ of kanamycin and rifampicin each) at 28°C. 1.5 mL of bacterial solution was centrifuged at 5 000 r min^-1^ for 3 min to collect bacterial cells. Then, the OD_600_ of agrobacterium suspension, including 1 mol L^-1^ MES (pH 5.6), 1 mol L^-1^ MgCl_2_ and 20 mmol L^-1^ acetosyringone, was adjusted to about 0.7. Agrobacterium suspension was standed still at 25°C for 1 h. The bacterial solution was slowly injected into the mesophyll cells on the back of Nicotiana benthamiana leaves, and the injected plants were cultured under light for 48 h. The subcellular localization of fusion protein was observed through LSM 710 NLO confocal microscope (Zeiss, Germany).

### 2.7 Statistical analysis

All the data are presented as mean ± SD (standard deviation). Microsoft Excel 2016 and SPSS17.0 were used to process the data while comparing the significance between treatments was done using the LSD method.

## 3 Results

### 3.1 Analysis of phenotypes, anthocyanin content and their components of different organs of mung bean

**Figure 1:**
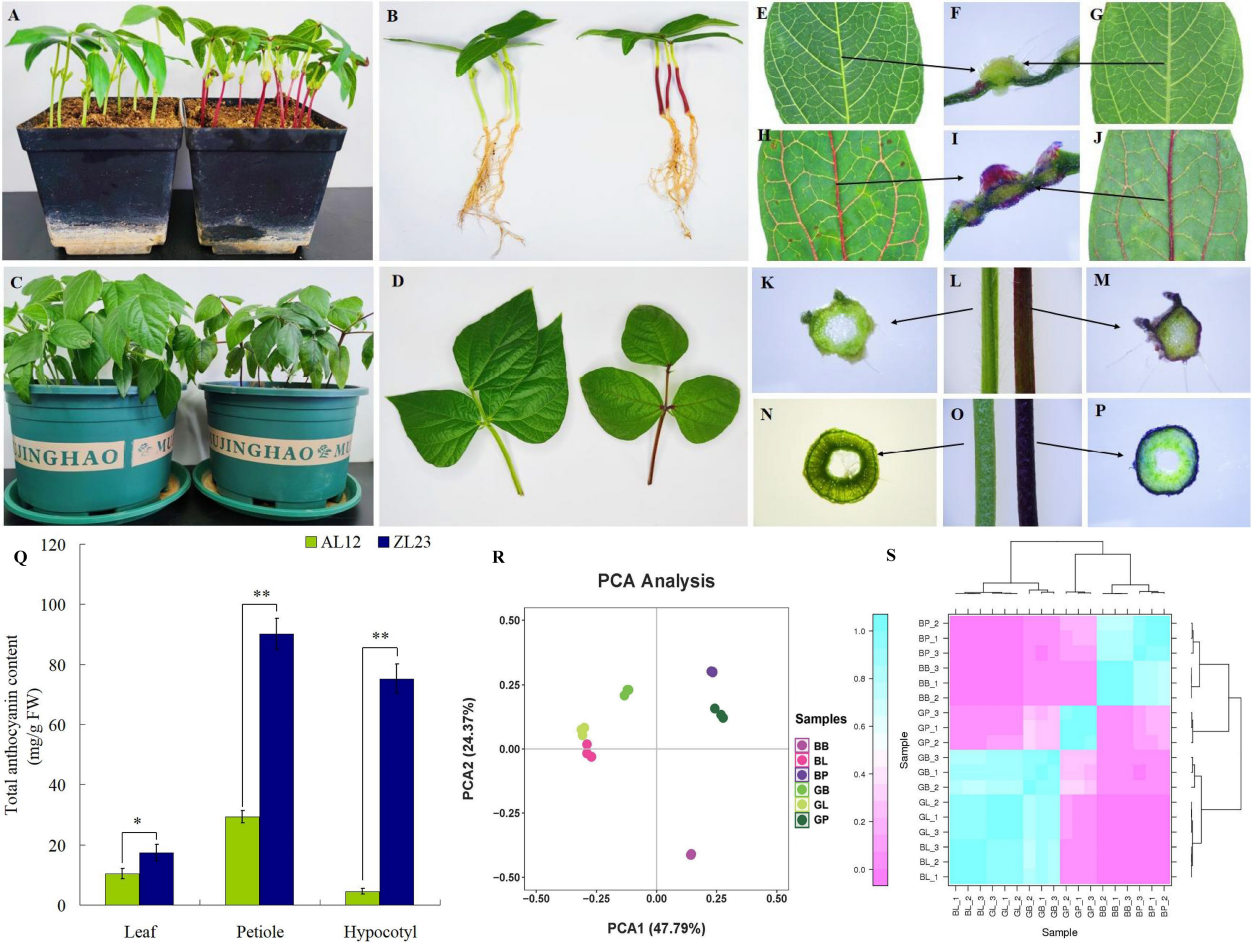
Phenotypes of AL12 (almost no anthocyanidin in the aboveground organs) and ZL23 (ZL23, anthocyanidin rich in leaves, petioles and hypocotyls) varieties of mung beans. **(A, B)** AL12 (left) and ZL23 (right) 10 days after sowing. **(C, D)** AL12 (left) and ZL23 (right) 20 days after sowing. **(E-G)** Leaf phenotype of AL12. **(H-J)** Leaf phenotype of ZL23. **(K)** Petiole crosscutting phenotype of AL12. **(L)** Petiole phenotype of AL12 (left) and ZL23 (right). **(M)** Petiole crosscutting phenotype of ZL23. **(N)** Hypocotyls crosscutting phenotype of AL12. **(O)** Hypocotyls phenotype of AL12 (left) and ZL23 (right). **(P)** Hypocotyls crosscutting phenotype of ZL23. **(Q)** Changes in total anthocyanin content in leaves, petioles and hypocotyls. The data were in the form of mean ± standard deviation. * and ** indicate significant difference at p< 0.05 and p< 0.01 levels, respectively. **(R)** PCA of metabolome data. **(S)** Metabolome data among three biological duplications. The x-axis represents principal component 1 (PC1); the y-axis represents principal component 2 (PC2); two cultivars (AL12 and ZL23) and three developmental organs (GB, GL, and GP stand for leaves, petioles, and hypocotyls of AL12; while for ZL23 as BL, BB, and BP, respectively) are distinguished by different colors.

To investigate the biosynthesis and accumulation of anthocyanins during the growth and development of AL12 and ZL23 mung bean varieties, the phenotype and anthocyanin content of leaves, petioles, and hypocotyls of both varieties were analyzed. All three parts in the common green bean variety were green in color with no anthocyanin accumulation observed during growth and development. In contrast, the leaf veins, petioles, and hypocotyls of the greenish-black bean leaves showed significant anthocyanin accumulation during the same period (**Figures 1A-P**). The anthocyanin content in leaves, petioles, and hypocotyls during the same period were found to be significantly lower in AL12 compared to ZL23. A substantial difference in anthocyanin contents among the two varieties was found in petioles and hypocotyls, where AL12 contained 29.3 mg g^-1^ in petioles and 4.5 mg g^-1^ in hypocotyls, while the same was found to be 90.2 mg g^-1^ and 75.2 mg g^-1^ in ZL23, respectively, showing a 3.07-fold and 16.7-fold increased anthocyanin contents in ZL23. Nevertheless, the difference in anthocyanin contents in leaves among the two varieties was relatively small i.e., 10.3 mg g^-1^ in AL12, and 17.4 mg g^-1^ in ZL23, respectively, where the contents in ZL23 were still 1.7-fold higher compared to AL12 (**Figure 1Q**), which could be attributed to the fact that anthocyanin in leaves is mainly accumulated in the leaf veins.

**Figure 2:**
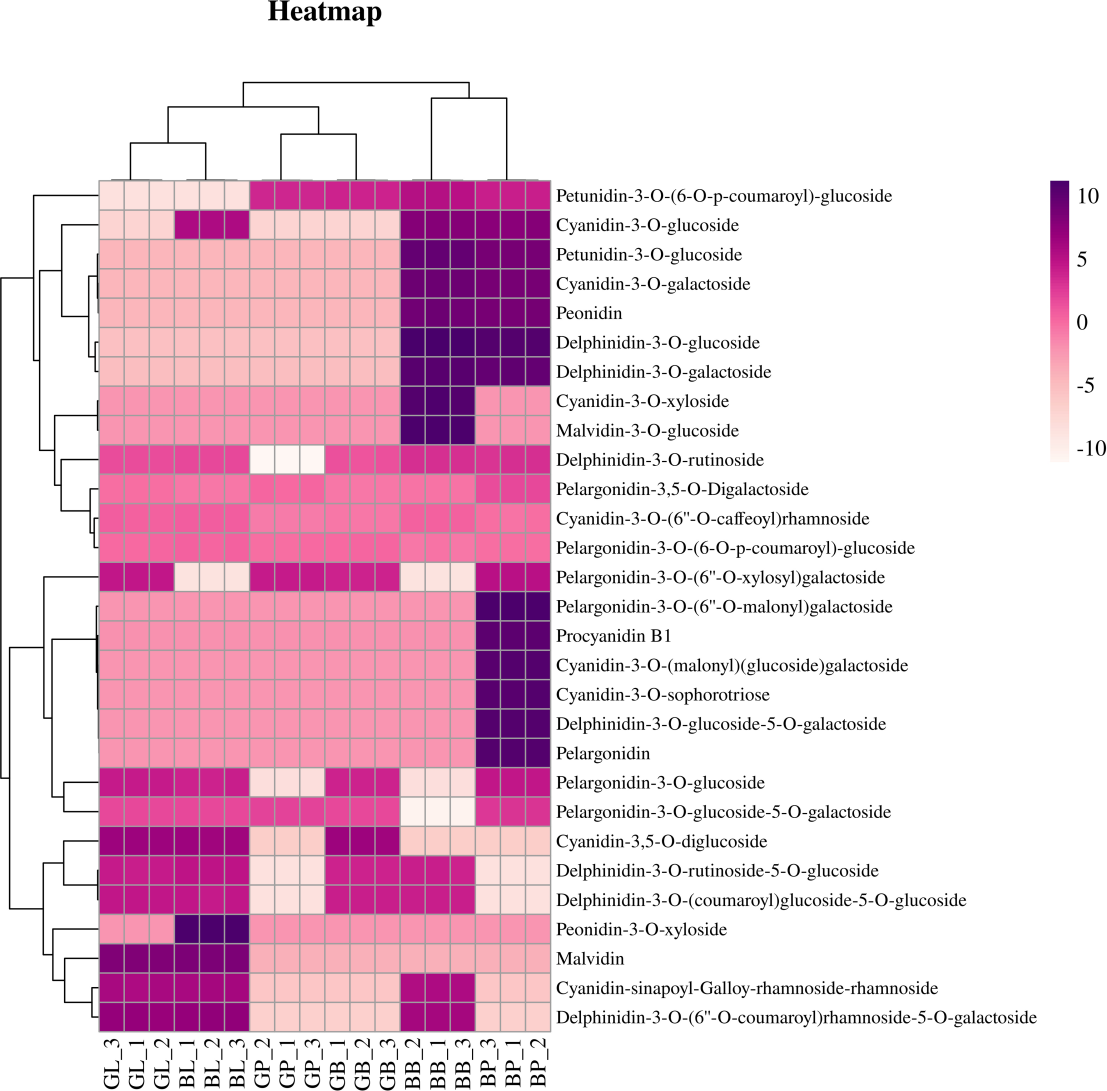
Hierarchical cluster analysis based on the anthocyanin components contents in mung bean.

Principal component analysis (PCA) showed that there were significant differences between the two varieties and three site samples (**Figure 1R**). 72.16% of the differences between samples could be explained by PCA1 (47.79%) and PCA2 (24.37%), indicating that anthocyanins showed a dynamic change pattern during two varieties and three site samples. Hierarchical cluster analysis (HCA) of metabolome data exhibited low difference in biological replicates. The correlation coefficient of anthocyanin components contents was up to 0.93 among biological replicates (**Figure 1S**). This suggests that there was a good correlation among the bio-replicates. The above results demonstrated the results was stable and repeatable, and provided a guarantee for the reliability of the results. Seven categories, consisted of 29 kinds of anthocyanin compounds, in these three stages were identified through LC-MS/MS, including 8 kinds of cyanidin, 7 kinds of delphinidin, 7 kinds of pelargonidin, 2 kinds of malvidin, 2 kinds of peonidin, 2 kinds of petunidin, and 1 kinds of procyanidin (**Figure 2**, **Table S2**). In AL12, cyanidin was the highest proportion of anthocyanins in the leaves and petioles, accounting for 84.1% and 55.9% respectively; pelargonidin was the highest proportion of anthocyanins in the hypocotyl, accounting for 82.8%. In ZL23, cyanidin was the highest proportion of anthocyanins in the leaves, accounting for 86.5%; delphinidin was the highest proportion of anthocyanins in the petioles and hypocotyl, accounting for 95.3% and 64.3%, respectively.

### 3.2 Global analysis of RNA-seq

**Figure 3:**
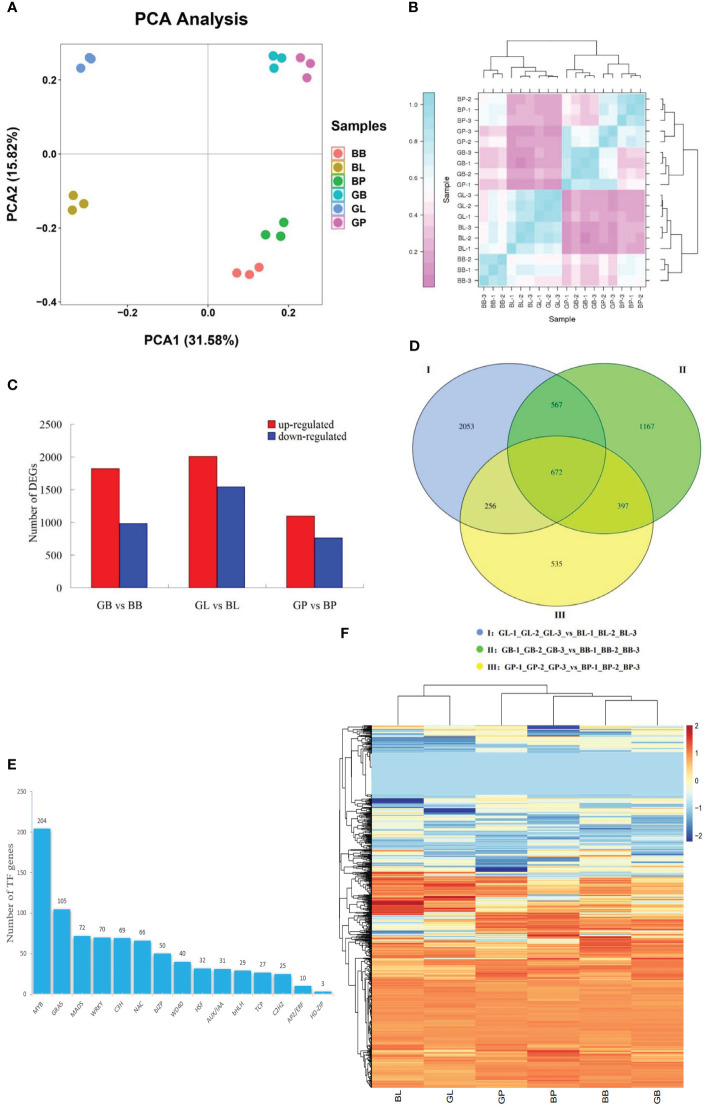
**(A)** PCA of transcriptome data. **(B)** transcriptome data among three biological duplications. The x-axis represents principal component 1 (PC1); the y-axis represents principal component 2 (PC2); two cultivars (AL12 and ZL23) and three developmental organs (GB, GL, and GP stand for leaves, petioles, and hypocotyls of AL12; while for ZL23 as BL, BB, and BP, respectively) are distinguished by different colors. **(C)** Number of differentially expressed genes in the same organs of AL12 and ZL23. **(D)** Wayne diagram of the number of differentially expressed genes between the same organs of AL12 and ZL23. **(E)** Number of transcription factors differentially expressed in AL12 and ZL23. **(F)** Hierarchical clustering analysis of the expression of each type of transcription factor in six different treatments (BB, BL, BP, GB, GL and GP).

The PCA results of transcriptome data showed a clear separation between the two varieties and three site samples, relatively. A negligible variability in transcriptome expression was found in GB and GP of AL12, and BB and BP of ZL23, while the same was higher in the leaves (GL, and BL). The 47.4% of the variation between samples was explained by PCA1 (31.58%) and PCA2 (15.82%), indicating that anthocyanin accumulation showed significant differences between the two varieties and different parts (**Figure 3A**). Correlation coefficient analysis between biological replicates as shown in **Figure 3B** indicated that the correlation coefficient of gene expression levels among biological replicates of all samples was >0.91, indicating an excellent reproducibility among biological replicates and that the data could be further used to determine DEGs.

To further clarify the molecular mechanism of anthocyanin biosynthesis during the growth and development of stated organs in ZL23, 18 cDNA libraries comprising two varieties of three organs in triplicates were prepared for transcriptome analysis. As shown in **Table S3**, a total of 116.64 Gb of clean data was obtained after filtering, with 19, 236, 210 to 38, 429, and 484 clean reads for each library. The percentage of Q30 (sequences with a sequencing error rate<0.1%) for each library was over 92%, while GC contents of the samples ranged between 44.67 and 46.20%. The clean reads were mapped between 83.58 and 93.87% to the reference genome of each library and which was then used for further analysis.

### 3.3 DEGs analysis

A total of 8211 DEGs were screened in the three comparison combinations as shown in **Figure 3C**, among which, a total of 4921 were up-regulated and 3284 were down-regulated. Additionally, 2803 DEGs were screened in the GB-1_GB-2_GB_3_vs_BB-1_BB_2_BB_3 comparison sample, among which 1882 were up-regulated and 981 were down-regulated. The GL-1_GL-2_GL_3_vs_BL-1_BL_2_BL_3 comparison sample screened a total of 3548 DEGs, with 2007 up-regulated and 1541 down-regulated genes, while GP-1_GP-2_GP_3_vs_BP-1_BP_2_BP_3 comparison sample screened a total of 1860 DEGs, where 1098 were up-regulated and 762 were down-regulated. These results suggested that the DEGs in AL12 and ZL23 might be involved in the synthesis of anthocyanins in mung beans. As shown in the Wayne diagram (**Figure 3D**), the number of DEGs specific to leaves, petioles, and hypocotyls were 2053, 1167, and 535, respectively, where the number of common DEGs between leaves and petioles was 567, and between leaves and hypocotyls was 256. Similarly, the number of common DEGs between petioles and hypocotyls was 397, while 672 were among leaves, petioles, and hypocotyls. Further, 833 transcription factors were found to be differentially expressed in the two varieties (**Figures 3E-F**, **Table S4**), belonging to 15 transcription factor families, among which 109 and 92 were significantly up- and down-regulated, respectively.

### 3.4 GO and KEGG analysis

The GO enrichment analysis results are shown in **Figure S1** and **Table S5**, where 52 functional components were grouped into 3 categories, including ‘biological processes’, ‘molecular functions’ and ‘cellular components’. The highest number of annotations was shown for ‘metabolic processes’ within ‘biological processes’, followed by ‘cellular processes’. In ‘cellular components’, the most numerous genes were annotated as ‘cellular’, ‘cellular components’, and ‘membrane components’. Similarly, in ‘molecular function’, the most genes were annotated as ‘binding’ and ‘catalytic activity’. These results indicated that many types of enzymatic pathways were active in mung beans. To further identify the metabolic pathways of DEGs, KEGG enrichment analysis was performed. As shown in **Figure S2**; **Table S6**, the top 20 KEGG pathways with the lowest Q values were mainly concentrated in ‘phytopathogen interaction’, ‘phenylpropane biosynthesis’, ‘photosynthesis’, ‘flavonoid biosynthesis’, and ‘flavonoid and flavonol biosynthesis’ pathways. The ‘flavonoid biosynthesis’ pathway was significantly enriched in all three comparative combinations, and results suggested that anthocyanin biosynthesis belonged to the flavonoid pathway and was also associated with the phenylalanine metabolic pathway. The large enrichment of the flavonoid biosynthesis pathway correlated with the large amount of anthocyanin synthesis in ZL23.

### 3.5 Analysis of structural and regulatory genes of anthocyanin synthesis in the different organs of mung bean

**Figure 4:**
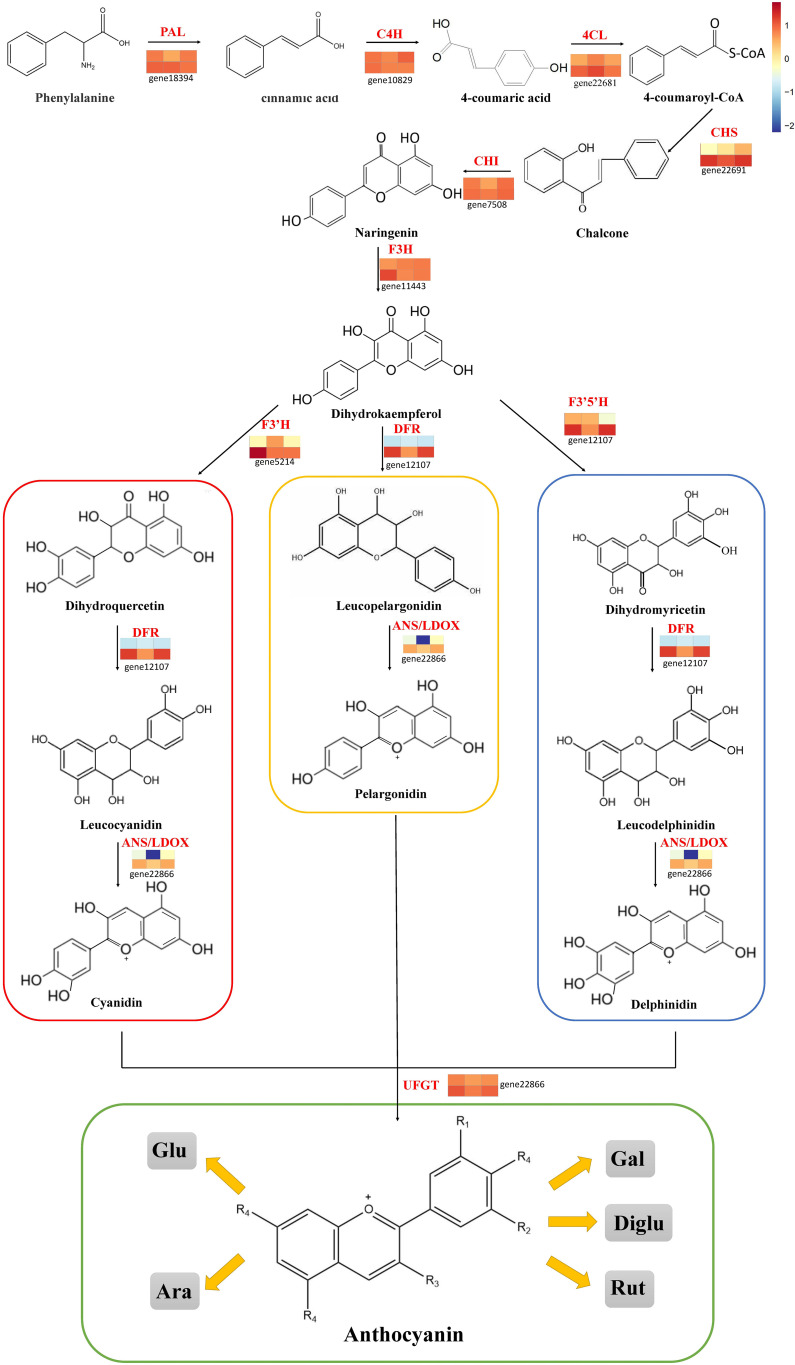
Transcript profiling of anthocyanin biosynthesis pathway in AL12 and ZL23.

**Figure 5:**
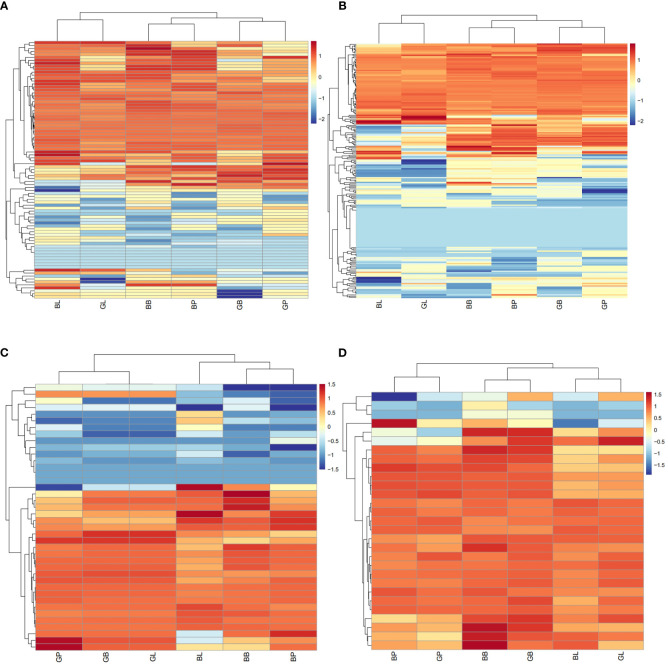
**(A)** HCA of structural genes for anthocyanin biosynthesis based on the RNA-Seq. **(B)** HCA of potential regulatory genes of MYB based on the RNA-Seq. **(C)** HCA of potential regulatory genes of bHLH based on the RNA-Seq. **(D)** HCA of potential regulatory genes of WD40 based on the RNA-Seq.

The published literature suggests that anthocyanins are mainly synthesized by 12 structural genes i.e., *CHI*, *CHS*, *C4H*, *CHS*, *4CL*, *DFR*, *F3*’*H*, *F3*’*5*’*H*, *F3H*, *LDOX*, *PAL*, *UFGT*, and three major transcriptional factors namely MYB, bHLH, and WD40, which were made into HCA (**Figures 4**, **5**). As shown by HCA results, the reliability of the anthocyanin content and PCA results was further illustrated by the fact that the genes related to anthocyanin synthesis were significantly different between the two varieties. The HCA of structural genes is shown in **Figure 5A**, which were at significantly higher expression levels in the petioles and hypocotyls of ZL23 compared to AL12. However, the differences in expression in leaves were relatively small. Moreover, the three regulatory genes i.e., *MYB*, *WD40*, and *bHLH* showed similar results as shown in **Figures 5B-D** and **Table S4**.

### 3.6 Validation of RNA-seq data by RT-qPCR

**Figure 6:**
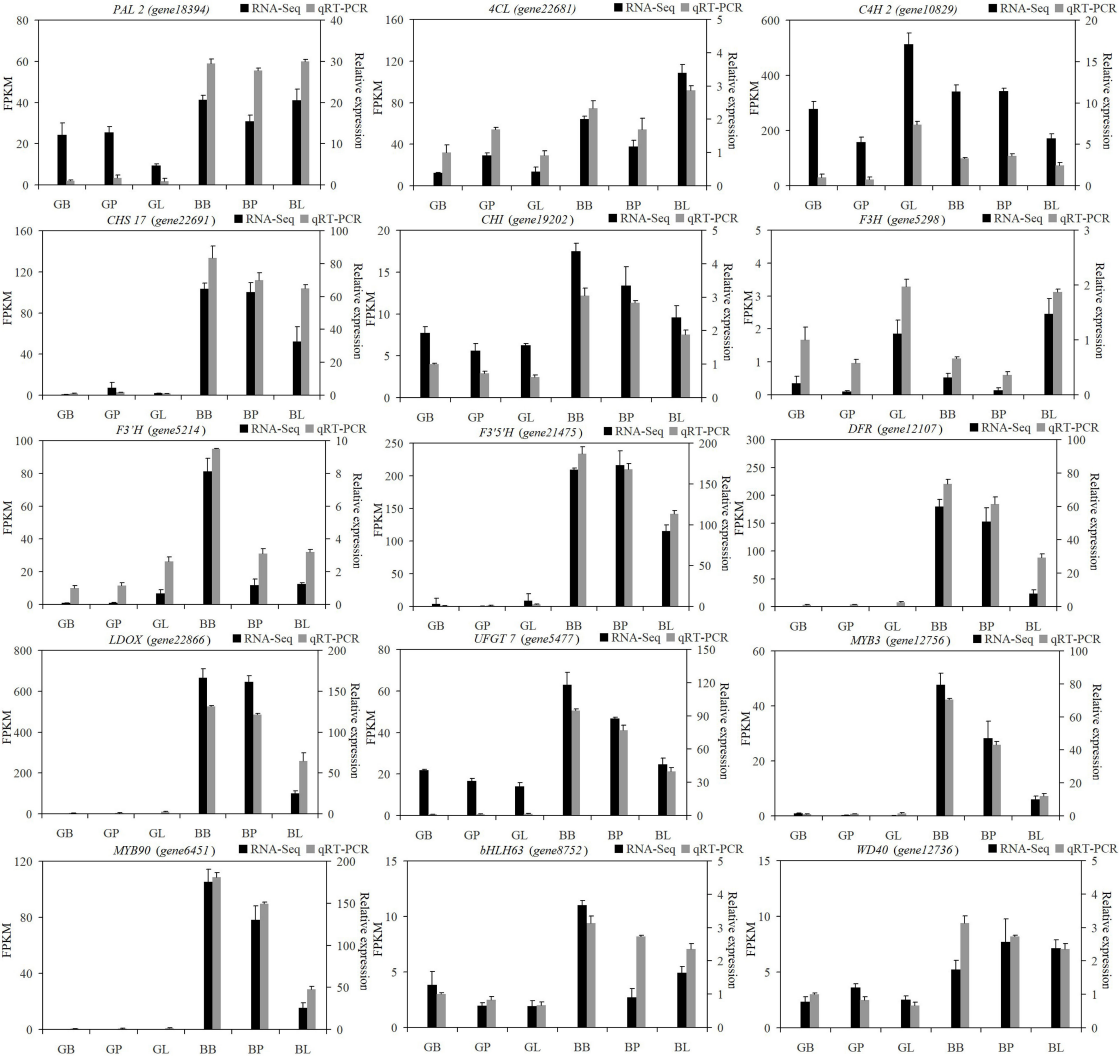
Expression analysis of fifteen genes by the RNA-Seq and qRT-PCR.

To further characterize the expression of genes related to anthocyanin biosynthesis, the relative expression of 12 structural genes for anthocyanin biosynthesis and *MYB*, *bHLH*, and *WD40* were determined by RT-qPCR (**Figure 6**). The expression profile results indicated a high correlation between RNA-seq and RT-qPCR, thereby further cementing the reliability of the RNA-seq results.

### 3.7 Gene Co-expression Networks in the different organs of mung bean

**Figure 7:**
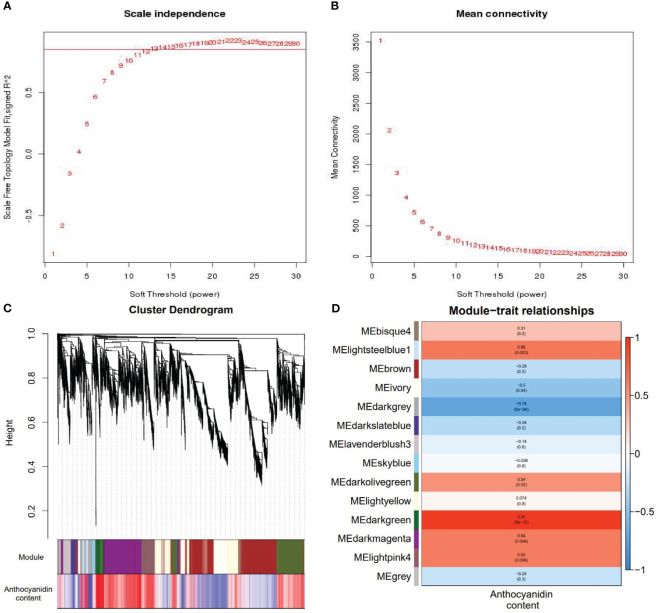
Weighted gene coexpression network analysis of metabolites and genes. **(A, B)** Analysis of network topology for various soft thresholding (power) and **(C)** construction of co-expression module. The same color represents the same module. If the module-features genes between two different modules are similar, they will be merged automatically. **(D)** Correlation heatmap between anthocyanin content and 14 gene modules. The y-axis represents each module. The x-axis represents anthocyanin content. Red and blue colors indicate up-regulated and down-regulated transcripts, respectively.

**Figure 8:**
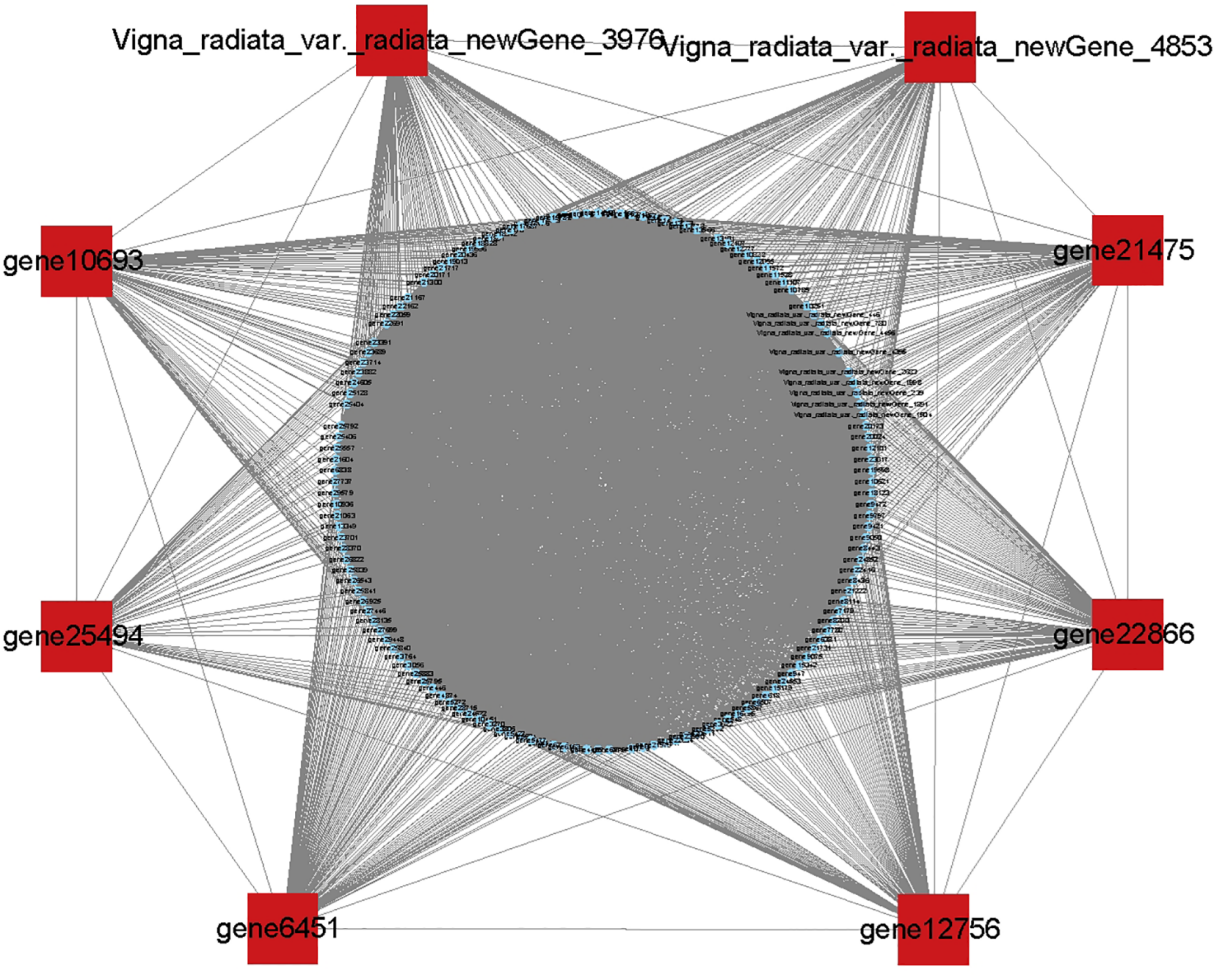
Based on the eight genes with the highest connectivity in the green module, the red dots are core genes and the blue dots are reciprocal genes.

To identify co-expressed gene modules and explore the relationship between gene networks and anthocyanin synthesis, the WGCNA was further performed, including 6415 DEGs. The correspondence between correlation coefficients and average connectivity at different thresholds (power values = 1 to 30) was measured. As shown in **Figure 7**, correlation coefficients, and average connectivity were guaranteed when the power value was 30. Finally, 14 modules were obtained from the clustering dendrogram (**Figure 7D**), where each module contained a different number of genes, with the ‘BROWN’ module containing the most genes at 1511 and the ‘BLUE’ module containing the least genes at 49, with an average of 458 genes per module. Pearson correlation analysis was performed between the genes and traits in each module according to the amount of anthocyanins in each site, and results are shown in **Figure 7**. The results demonstrated that seven of the 14 modules were positively correlated and seven were negatively correlated with anthocyanin synthesis. The green and dark-grey modules had the highest positive and negative correlation with anthocyanin content, respectively. The top 8 genes in the green module were selected as the core genes, including Vigna_radiata_var._radiata_newGene_3976, Vigna_radiata_var._radiata_newGene_4853, gene10693, gene25494, gene6451, gene12756, gene22866, and gene21475. These genes and their associated genes were mapped in a visual gene interaction network (**Figure 8**). Results showed that gene22866 and gene21475, which encode LDOX and F3’5’H respectively, were structural genes for anthocyanin synthesis, while Vigna_radiata_var._radiata_newGene_3976, Vigna_radiata_var._radiata_newGene_4853, gene10693, gene25494, gene6451, gene12756 belonged to MYB transcription factors. Due to the low expression of Vigna_radiata_var._radiata_newGene_3976, Vigna_radiata_var._radiata_newGene_4853, gene10693, gene25494 (FPKM ≤10), they will be ignored. Based on the combined analysis of the above results, gene12756 (*VrMYB3*) and gene6451 (*VrMYB90*) were found to play important roles in the regulation of anthocyanin synthesis in mung beans.

### 3.8 Subcellular localization analysis of VrMYB3 and VrMYB90

**Figure 9:**
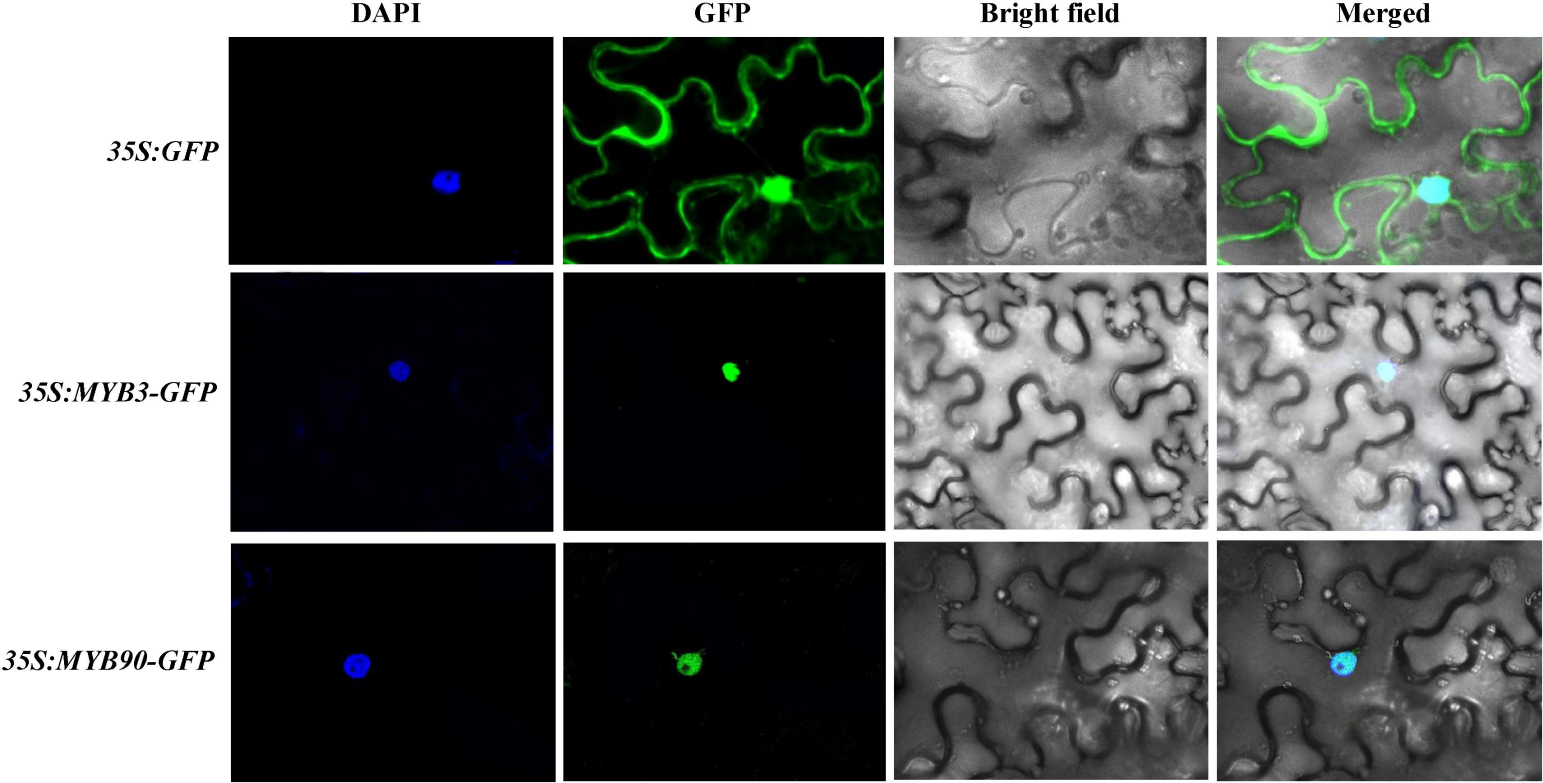
Subcellular localization analysis of VrMYB3 and VrMYB90 in *N. benthamiana* cells.

To confirm the location of VrMYB3 and VrMYB90 proteins in cells, the transiently expressed fusion proteins of VrMYB3 and VrMYB90 in tobacco leaves were detected by confocal laser microscopy. **Figure 9** showed that there were GFP signals on both cell nucleus and cell membrane after transiently expressed *35S:GFP* vector in tobacco epidermal cells. However, transiently expressed of *35S:VrMYB3-GFP* and *35S:VrMYB30-GFP* vectors in tobacco epidermal cells, GFP signals were present on the nucleus. It suggests that VrMYB3 and VrMYB90 have potential function of transcriptional regulatory.

## 4 Discussion

Anthocyanins confer plants’ colorful phenotype, increase their attractiveness to insects, and improve pollination efficiency, as well as improve resistance to adverse conditions and survival ability (Liu et al., 2020). They are water-soluble pigment found in plants and are generally prevalent in all types of plant organs. However, they usually stored as more stable glycosides in the vesicles of the plant’s epidermal cells, accounting for the wide range of colors in plant organs (Li et al., 2021). For most plants, anthocyanins often accumulate in light-exposed locations e.g., in the seed coat of wheat (Flores et al., 2022) and maize (Paulsmeyer et al., 2022), the dextrin layer of rice (Peanparkdee et al., 2020), the seed coat of mung beans (Ma et al., 2023), etc. Moreover, some plants can also accumulate in other above-ground parts, e.g., rice (Mackon et al., 2021), wheat (Zhao et al., 2019), and maize (Lopez-Malvar et al., 2017), which suggests that light is an essential instigator for the synthesis of plant anthocyanins. Interestingly, underground plant organs such as purple potatoes, purple sweet potatoes, and black peanuts are also able to accumulate anthocyanins in large quantities (Kuang et al., 2018; Li et al., 2019a), suggesting anthocyanin accumulation is regulated by different genes in different species. In this study, the results indicated that the ZL23 mung bean variety accumulated anthocyanins mainly in the leaf veins, petioles, and hypocotyls. Though it accumulated anthocyanins in significant quantities in the above-ground plants, its seed coat remained normal for mung beans, implying the involvement of a complex regulatory mechanism for anthocyanin accumulation in mung beans. In the current study, the components and contents of anthocyanins in two varieties and three site samples were identified by using liquid chromatography-tandem mass spectrometry. A total of 7 categories and 29 kinds of anthocyanins were detected. The content of most components contents were significantly higher in ZL23 compare with AL12. Interestingly, the main components of anthocyanins in three site samples were not the same. For example, in the petioles and petioles of ZL23, delphinidin-3-O-glucoside was the highest proportion of anthocyanins, accounting for 95.3% and 64.3%, respectively; however, in the leaves, cyanidin-3-O-(6’’-O-caffeoyl)-rhamnoside was the highest proportion of anthocyanins, accounting for 86.5%. It suggested that the components and contents of anthocyanin varied greatly among different organs or different varieties of the same crop.

The synthesis of anthocyanins is regulated by two main groups of genes i.e., structural genes, which promote the synthesis of anthocyanins from phenylalanine by regulating a series of enzymatic reactions of phenylalanine in plants, and regulatory genes regulating anthocyanin biosynthesis by inducing or inhibiting the transcriptional levels of structural genes (Li et al., 2022c). Currently, the structural genes for anthocyanin synthesis have been relatively well studied and their biological functions were relatively clear, including *PAL*, *CHS*, *CHI*, *4CL*, *C4H*, *LDOX*, *F3H*, *F3’H*, *F3’5’H*, *DFR*, *ANS*, *UFGT*, etc (Chen et al., 2020). Similarly, a significant number of studies have focused on *MYB*, *WD40*, and *bHLH* for regulatory genes (Sharma et al., 2020). In this study, 26 structural and 78 regulatory genes were found to be differentially expressed in both varieties. Further analysis revealed that some structural and regulatory genes were significantly correlated with anthocyanin content. In addition, other transcription factors that may be involved in the regulation of anthocyanin synthesis were also identified, e.g., 18 *bZIPs*, 20 *NACs*, and 9 *MADSs* were differentially expressed in the two mung bean varieties, respectively. Notably, many studies have identified regulatory genes beyond the MYB-bHLH-WD40 ternary complex e.g., peach *PpNAC1* (Zhou et al., 2015), tomato *SlHY5* (He et al., 2023), and sweet potato *IbMADS10* (Dong et al., 2016) are all involved in the regulation of anthocyanin biosynthesis. These data suggest that plant anthocyanin biosynthesis is controlled by a complex and sophisticated regulatory network.

With the development of sequencing technology, the systematic study of massive amounts of genomic, transcriptomic, and metabolomic data using co-expression networks has gained immense attention for data processing (Xie et al., 2022). The WGCNA is an effective method for co-expression network analysis, capable of specifically screening out co-expression modules with high biological significance to the target trait, and has proven to be an efficient data mining method in a variety of plants (Sahu et al., 2019; Lu et al., 2019). In traditional methods, differential trait comparison is often focused on finding differential genes, thus neglecting the inter-genes correlation. Many genes have similar expression patterns and may have similar functions, allowing similar inter-genes interactions that regulate similar biological metabolic processes or the protein products they produce (Li et al., 2023). Through WGCNA, the clustering of genes with similar expression patterns can be used to identify co-expressed gene modules, explore the biological relevance of the modules to the target traits, and mine the core genes in the network (Wan et al., 2020). In this study, a weighted gene co-expression network was constructed by WGCNA on 18 sets of transcriptome sequencing data from the two mung bean varieties, and co-expression modules were identified that were highly significantly associated with anthocyanin expression i.e., Medarkgreen (0.97), Melightsteelblue1 (0.66), Medarkmagenta (0.64), and Melightpink4 (0.62), where Medarkgreen module showed strong correlation. The genes in the Medarkgreen module were constructed as a visual gene interaction network map, and the top eight genes with the highest gene connectivity within the module were selected as hubgene, among which, gene22866 (*LDOX*, kME=0.9673) and gene21475 (*F3’5’H* kME=0.9709) were identified as structural genes for anthocyanin biosynthesis, while Vigna_radiata_var._radiata_newGene_3976 (*MYB*, kME=0.9685), vigna_radiata_var._radiata_newGene_4853 (*MYB*, kME= 0.9756), gene10693 (*MYB4*, kME=0.9696), gene25494 (*MYB*, kME=0.9642), gene6451 (*MYB90*, kME=0.9713), and gene12756 (*MYB3*, kME=0.9627) were found to be MYB transcription factors. These results are envisaged to indicate that the Medarkgreen module was highly associated with anthocyanin biosynthesis in ZL23. Therefore, it was speculated that the above genes may have important regulatory roles in the synthesis of anthocyanins in mung beans.

In addition to the direct involvement of structural genes, the process of anthocyanin synthesis was regulated by a protein complex formed by MYB, WD40, and bHLH, with major involvement of MYB. The MYB transcription factors can be further classified based on the number of structural domains into MYB-related, R2R3-MYB, R1R2R3-MYB, and atypical MYB, R2R3-MYB, R1R2R3-MYB, and atypical MYB, among which R1R2R3-MYB was mainly found in animals, and R2R3-MYB in plants, in addition to abundant R2R3-MYB (Shui et al., 2022). The number of atypical MYBs was relatively small and needs further exploration. A study reported that out of nearly 200 MYB *Arabidopsis* transcription factors, there were 126 R2R3-MYB class transcription factors (Li et al., 2023). Similarly, among the nearly 200 reported MYBs in rice, 109 were R2R3-MYBs. These proteins are reported to contain two MYB domains (R2, R3) at the N-terminus and are widely involved in the synthesis of plant secondary metabolites, as well as in response to various stressors and stress responses (Chen et al., 2006). R2R3-MYB transcription factors are the most important regulatory factors in the anthocyanidin biosynthesis pathway known to date. The type and content of anthocyanin were determined by regulating the expression of the structural gene of anthocyanin biosynthesis, which ultimately affects the colors of flowers, fruits, and leaves (Yin et al., 2021). The R2R3-MYB family has been further divided into 25 subfamilies based on the different conserved amino acid sequences of R2R3-MYB proteins, among which some have been shown closely related to anthocyanin biosynthesis e.g. (Zhou et al., 2020), in *Arabidopsis thaliana*, subfamily 5 AtMYB123 was involved in the accumulation of procyanidine in seeds coat, 6 AtMYB75, AtMYB90, AtMYB113, and AtMYB114 regulated the synthesis of anthocyanins in nutritional tissues, and 7 AtMYB11, AtMYB12, and AtMYB111 regulated the synthesis of anthocyanins in all *Arabidopsis* organs. In addition, most members of subclade 4 were negative regulators of the anthocyanin synthesis pathway in Arabidopsis. Our results suggested that MYB3 and MYB90, the R2R3-MYB transcription factor family members, increased the anthocyanin content by regulating the expression of structural genes such as *PAL*, *4CL*, *F3’5’H*, *LDOX*, and *F3’H*. Further, our data present a potential working model for elucidating the molecular mechanism of mung bean anthocyanin synthesis (**Figure S3**), however, how MYB3 and MYB90 precisely control anthocyanin synthesis required further investigation.

## 5 Conclusions

This study compared two different mung bean varieties with significantly different anthocyanin accumulation as test material. High-throughput sequencing analysis results showed that anthocyanin accumulation in ZL23 was mainly concentrated in the leaf veins, petioles, and hypocotyls. Transcriptome combined with WGCNA analysis indicated MYB3 and MYB90 were responsible for increasing anthocyanin content by inducing the expression of structural genes such as anthocyanin *PAL*, *4CL*, *F3’5’H*, *LDOX*, and *F3’H*. Our study further suggests that the nuclear localization of MYB3 and MYB90 were highly correlated with anthocyanin biosynthesis, which is envisaged to play a pivotal regulatory role in the biological process of anthocyanin biosynthesis in mung beans. It is envisaged to further enhance the current understanding of anthocyanin biosynthesis in mung beans and provide valuable information for breeding anthocyanin-rich cereals.

## Data availability statement

The datasets presented in this study can be found in online repositories. The names of the repository/repositories and accession number(s) can be found below: BioProject, PRJNA991706.

## Author contributions

CL: conceptualization, methodology, data curation, writing-original draft preparation, writing-review and editing, visualization, supervision and funding acquisition. ZG: conceptualization and writing-original draft preparation. WH: software and validation. XZ: software and validation. YL: investigation. NL: investigation. CM: conceptualization, methodology, data curation, writing-review and editing, visualization, supervision, project administration and funding acquisition. All authors contributed to the article and approved the submitted version.

## Supplementary material

Supplementary Figure 1
**(A-C)** Plots of GO enrichment analysis of differentially expressed genes in the combination (GBvsBB, GLvsBL and GPvsBP) of AL12 and ZL23.

Supplementary Figure 2
**(A-C)** KEGG pathway from TSA enriched in the combination (GBvsBB, GLvsBL and GPvsBP) of AL12 and ZL23. y-axis indicates the KEGG pathway; x-axis indicates the enrichment fraction.

Supplementary Figure 3 A possible molecular mechanism model for anthocyanin biosynthesis in mung bean.
