# Supplementary figures and images for: Integration of comparative transcriptomics and WGCNA characterizes the regulation of anthocyanin biosynthesis in mung bean (Vigna radiata L.)
Source: Front Plant Sci. 2023 Oct 24;14:1251464. doi: 10.3389/fpls.2023.1251464 (PMC10628539; doi:10.3389/fpls.2023.1251464)

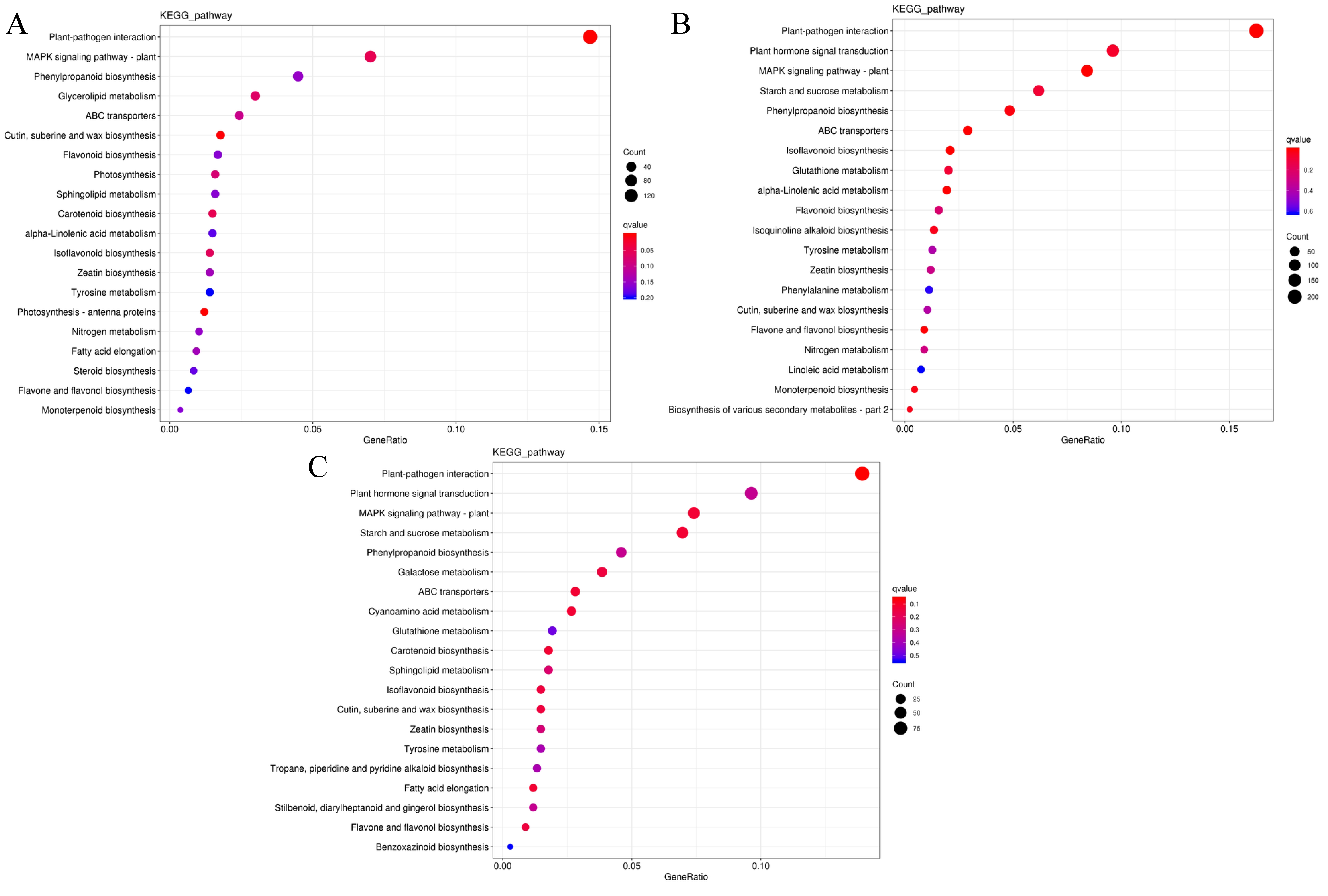

Supplement: Supplementary Figure 1 — (A-C) Plots of GO enrichment analysis of differentially expressed genes in the combination (GBvsBB, GLvsBL and GPvsBP) of AL12 and ZL23. [file Image_1.jpeg]

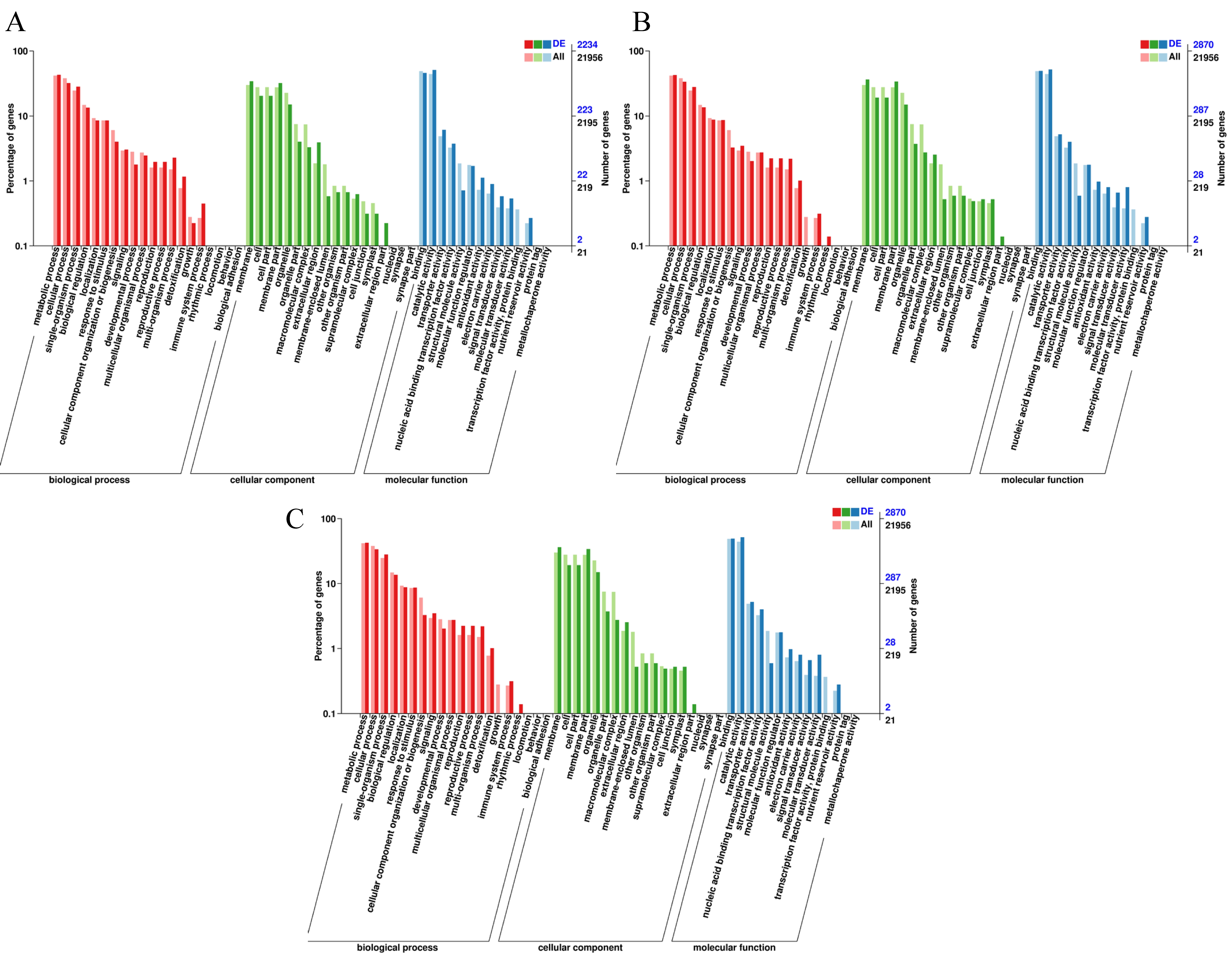

Supplement: Supplementary Figure 2 — (A-C) KEGG pathway from TSA enriched in the combination (GBvsBB, GLvsBL and GPvsBP) of AL12 and ZL23. y-axis indicates the KEGG pathway; x-axis indicates the enrichment fraction. [file Image_2.jpeg]

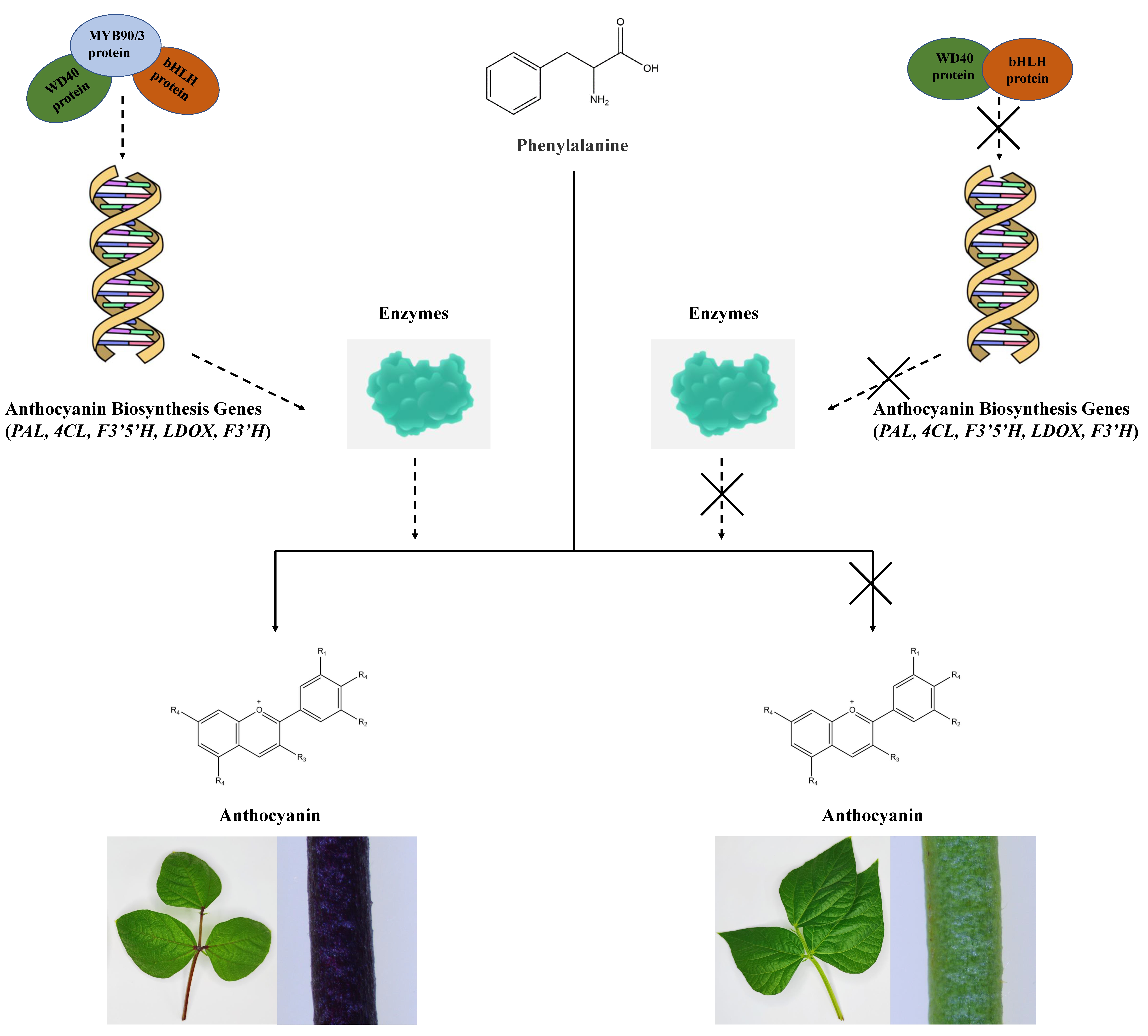

Supplement: Supplementary Figure 3 — A possible molecular mechanism model for anthocyanin biosynthesis in mung bean. [file Image_3.jpeg]
